# Formononetin protects against acetaminophen-induced hepatotoxicity through enhanced NRF2 activity

**DOI:** 10.1371/journal.pone.0170900

**Published:** 2017-02-24

**Authors:** Fen Jin, Chunpeng Wan, Weifang Li, Liangliang Yao, Hongqian Zhao, Yuan Zou, Dewei Peng, Weifeng Huang

**Affiliations:** 1 Medical College, China Three Gorges University, Yichang, Hubei, China; 2 Jiangxi Key Laboratory for Postharvest Technology and Nondestructive Testing of Fruits & Vegetables, College of Agronomy, Jiangxi Agricultural University, Nanchang, Jiangxi, China; 3 The Affiliated Hospital of Jiangxi University of Traditional Chinese Medicine, Nanchang, Jiangxi, China; University of South Alabama Mitchell Cancer Institute, UNITED STATES

## Abstract

To examine the effects of formononetin (FMN) on Acetaminophen (APAP)-induced liver injury *in vitro* and *in vivo*. Human non-tumor hepatic cells LO2 were pretreated with either vehicle or FMN (20, 40 μM), for 6 h, followed by incubation with or without APAP (10 mM) for 24 h. In an *in vivo* assay, male BALB/c mice were randomly divided into four groups: (1) control group; (2) APAP group; (3) APAP + FMN (50 mg/Kg); (4) APAP + FMN (100 mg/Kg). The mice in the control and APAP groups were pre-treated with vehicle; the other two groups were pretreated daily with FMN (50, 100 mg/Kg) orally for 7 consecutive days. After the final treatment, acute liver injury was induced in all groups, except the control group, by intraperitoneal (i.p.) injection of 300 mg/Kg APAP. In LO2 cells, APAP exposure decreased the cell viability and glutathione (GSH) content, which were both greatly restored by FMN pretreatment. Overdose of APAP increased hepatic malondialdehyde (MDA) content, serum alanine aminotransferase (ALT), and aspartate aminotransferase (AST) activity in experimental mice. Supplementation with 100 mg/Kg FMN significantly reduced APAP-induced elevated levels of MDA (1.97 ± 0.27 *vs* 0.55 ± 0.14 nmol/mg protein, *p* < 0.001), ALT (955.80 ± 209.40 *vs* 46.90 ± 20.40 IU/L, *p* < 0.001) and AST (1533.80 ± 244.80 *vs* 56.70 ± 28.80 IU/L, *p* < 0.001), and hepatic GSH level (5.54 ± 0.93 *vs* 8.91 ± 1.11 μmol/mg protein, *p* < 0.001) was significantly increased. These results were further validated by histopathology and TdT-mediated biotin-dUTP nick-endlabeling (TUNEL) staining, pretreatment with 100 mg/Kg FMN significant decreased APAP-induced hepatocellular damage and cell apoptosis (36.55 ± 3.82 *vs* 2.58 ± 1.80%, *p* < 0.001). Concomitantly, FMN stimulated the expression of Nrf2 and antioxidant gene expression in the presence of APAP. These data provide an experimental basis for the use of FMN in the treatment of patients with APAP-induced hepatotoxicity.

## Introduction

Acetaminophen (APAP, also known as paracetamol, N-acetyl-p-aminophenol and Tylenol^®^), is a nonsteroidal analgesic and antipyretic drug that is clinically widely used. At therapeutic doses, APAP is metabolized to water-soluble metabolites in the liver by UDP-glucuronosyltransferases (UTGs) and sulfotransferase (SULTs), with only a small amount being converted by cytochrome P450 (CYP450) 2E1 into the highly reactive, cytotoxic intermediate *N*-acetyl-*p*-benzoquinoneimine (NAPQI)[1–3]. The small amounts of NAPQI generated are normally effectively detoxified by glutathione (GSH) conjugation and cleared from the liver[4]. However, excessive intake of APAP saturates the glucuronidation and sulfation routes, resulting in the formation of large a mounts of NAPQI converted by CYP450, which is then detoxified by conjugation with GSH. Once GSH is depleted, NAPQI covalently binds to possibly critical cellular proteins, with formation of reactive oxygen species (ROS), causing hepatocellular necrosis and apoptotic cell death, eventually resulting in severe centrilobular hepatotoxicity and acute liver failure[5–7].

The transcription factor, nuclear factor erythroid 2 p45-related factor 2 (Nrf2), regulates the expression of a wide array of genes involved in GSH synthesis, antioxidative system, drug metabolism via binding to the antioxidant response element (ARE) for hepatoprotection[8]. There are several pieces of evidence to corroborate the fact that Nrf2-mediated gene regulation is active in the protection of APAP-induced hepatotoxicity. After activation of Nrf2 in mice deficient for kelch-like ECH associated protein 1 (keap 1), an Nrf2 inhibitor protein, were more resistant to toxic doses of APAP[9]. Conversely, in Nrf2-deficient mice, the higher susceptibility to APAP leads to greater severity in hepatic damage and increased lethality[9, 10].

As is known to all, traditional Chinese herbs are significant sources of drugs for the treatment of diseases. Red clover isoflavones are used in highly concentrated food supplements as an alternative to hormone replacement therapy, which has a long history of medicinal use, and possesses anti-inflammatory, antioxidant and anti-apoptosis activity[11, 12]. Next, Recent studies have demonstrated that red clover isoflavones can be used to alleviate the side-effects of drugs[13]. Furthermore, red clover isoflavones enriched with formononetin (FMN) have been reported to lower serum LDL cholesterol in a randomized, double-blind, placebo-controlled study[14]. FMN is one of the major isoflavone constituents isolated from red clover, which has been demonstrated diverse pharmacological benefits, and which serves as a source of potential therapeutic compounds for disease treatment[15, 16]. Accumulating evidences has demonstrated the anticancer activity of FMN in breast cancer and prostate cancer[17, 18]. However, the effect of FMN on APAP-induced liver injury has never been investigated.

The aim of this study was to explore the effect of FMN on the prevention of APAP induced hepatotoxicity, with emphasis on Nrf2 activity trying to elucidate the mechanisms by which FMN may execute its protective effects.

## Materials and methods

### Material and equipment

Dulbecco's modified eagle medium (DMEM) and fetal bovine serum (FBS) were purchased from Gibco, Grand Island, NY, USA. The FMN, APAP and 3-(4,5-dimethylthiazol-2-yl)-2,5-diphenyltetrazolium bromide (MTT) were purchased from Sigma Aldrich, Saint Louis, MO, USA. TdT-mediated biotin-dUTP nick-endlabeling (TUNEL) kit was purchased from Roche Diagnostics Gmbh, Mannheim, Germany. The kits for determining GSH and MDA contents were obtained from Jianchen, Nanjing, China. The kits for determining serum AST and ALT activity were obtained from Jianchen, Nanjing, China. Antibodies against Nrf2 (Cat# ab31163) were purchased from Abcam, Cambridge, MA and anti-β-actin (Cat#A1978) antibodies were from Sigma Aldrich, Saint Louis, MO, USA. BCA protein assay kit and enhanced chemiluminescent substrate kit were obtained from Pierce, Thermo Scientific, Rockford, IL, U.S.A. Protease inhibitor cocktail was purchased from KeyGen, Shanghai, China. Other reagents were of analytical grade.

### Cell culture and APAP treatment

Human non-tumor hepatic LO2 cells were obtained from the Chinese Academy of Sciences, Shanghai, China. Cells were maintained in DMEM medium supplemented with 10% FBS and incubated in a humidified incubator at 37°C in 5% CO_2_. The treatment in cell culture was initiated by replacing old media with a serum-free medium supplemented with or without FMN (20, 40 μM) for 6 h. Then, the cells were further incubated for APAP 10 mM for another 24 h. Cells collected were washed twice with PBS and used for cell viability or GSH analyses.

### Experimental animals and treatment

Male BALB/c mice, 6–8 weeks old weighing approximately 18–25 g, were purchased from China Three Gorges University Laboratory Animal Center. All animals were maintained under specific pathogen-free conditions at controlled temperature (20–25°C), humidity (45 ± 5%), and 12 h light/dark cycles in the China Three Gorges University Laboratory Animal Center. The animals were kept in sterile cages (maximum of 5 per cage) and fed standard rodent chow and allowed free access to water *ad libitum*. Animal husbandry was provided by the staff of China Three Gorges University Laboratory Animal Center under the guidance of supervisors who are certified Animal Technologists. All experiments were conducted using humane care in accordance with NIH Guidelines for the Care and Use of Laboratory Animals. Animal care and use was approved by the Experimental Animal Management Committee of China Three Gorges University (permit number: 2014110A).

Oral gavage was undertaken using flexible tubes which are less likely to cause oesophageal trauma. An animal’s ability to drink water, feed, and ambulate as well as its general appearance were evaluated after gavage. They were monitored third a day on the first day of gavage, daily during the following six days, and 3 times in 24 hours after APAP treatment. A new needle was used for each animal to reduce discomfort and the risk of any injection-site infection, injecting fluid at body temperature was used to prevent discomfort further. According the requirement of Experimental Animal Management Committee of China Three Gorges University, if the animal became severely ill in experimental process, the animal must be humanely killed by CO2 inhalation to prevent animal suffering. The monitoring criteria is: (1) inadvertent dosing into lung diagnosed by immediate signs of respiratory distress; (2) injection-site infection diagnosed by swelling or edema; (3) pain diagnosed by body movement and gesture; (4) body weight loss; (5) neurological defects, abnormal gait, hemiplegia or coma. Experimental mice liver tissue and blood was used in the present research. So, all the mice in the study were euthanized at the end of the experiment.

Mice were randomly divided into four groups (4–6 mice/group): (1) Control; (2) APAP; (3) APAP + FMN (50 mg/kg); (4) APAP + FMN (100 mg/kg). FMN powder was dissolved in 0.5% CMC-Na (sodium carboxyl methyl cellulose). APAP + FMN groups were pre-administered intragastrically with various doses of FMN for seven consecutive days, respectively. After the final treatment, APAP and APAP + FMN groups were given a single dose of APAP (300 mg/kg, i.p.) dissolved in phosphate-buffer saline (PBS, PH 7.4). Control mice were injected with equal volume of the vehicle. The animals were sacrificed by cervical dislocation under isoflurane anesthesia for the collection of blood (from vena cava) and a liver sample after 24 h of acetaminophen challenge. A portion of liver was cut and fixed in 4% paraformaldehyde, and the remaining parts were immediately stored at −80°C.

### Cell viability

Cell viability was determined by the MTT assay. After the treatment, 20 μL of MTT solution (5 mg/mL in PBS) was added to cells in 96-well plates and allowed to incubate for 4 h. The medium was aspirated and replaced with 200 μL/well of DMSO to dissolve the formazan salt formed. The plates were measured at 490 nm using a microplate spectrophotometer. The optical intensity of the formazan solution reflects the cell growth condition.

### Hepatocellular GSH

LO2 cells or liver tissues (~100 mg) were homogenized in 1 mL of ice-cold PBS containing 1mM EDTA (pH 7.5). The supernatants obtained from the cell or tissue homogenization by centrifugation at 10,500 ×g for 10 min at 4°C. Cellular protein content was determined by the BCA assay kit for cell or tissue homogenization before being used for the determination of GSH content using a GSH quantification kit (Jianchen, Nanjing, China) per the manufacturer's instructions.

### Measurement of intracellular ROS

After the treatment, cells were washed and resuspended in PBS. DCFH-DA (Beyotime, Nanjing, China) was then added to the suspended cells at a final concentration of 10 μmol/L in the dark in an incubator for 30 min and immediately used for ROS detection by flow cytometry at an excitation/emission wavelength of 485/530 nm. Results were also expressed as the percentage increase relative to untreated cells.

### Hepatic lipid peroxidation (MDA) assay

Lipid peroxidation of liver in mice was evaluated by measuring the thiobarbituric acid (TBA) according to the modified method by Ohkawa et al[19]. Liver tissue (~ 100 mg) was homogenized in 1 mL PBS containing 1 mM EDTA and centrifuged at 10,500 ×g for 10 min at 4°C. MDA content was determined by MDA quantification kit (Jianchen, Nanjing, China) per the manufacturer's instructions. MDA value was normalized to the hepatic cell protein content as determined by the BCA kit. The amount of lipid peroxidation was expressed as nmol/mg protein.

### Serum analyses

Blood obtained from the mice was placed at room temperature for 60 min to clot. After centrifugation at 4000×g for 15 min at 4°C, the serum was collected in new tubes. Serum AST and ALT activities were determined with the AST and ALT Test Kits from Jianchen (Nanjing, China).

### Western blot

Mice liver lysates were prepared with RIPA lysis buffer followed by centrifugation. Western blot analysis of Nrf2 in mice liver was performed per the standard protocols. Antibodies against Nrf2 were used at a dilution of 1:3000 or antibodies against β-actin at a dilution of 1:10000. The blots were detected by chemiluminescence followed by exposure to Kodak-X-Omat film (Shanghai, China).

### Quantitative real-time RT-PCR analyses

Total RNA was isolated from hepatocytes using the TRIzol reagent per manufacturer’s instructions (Invitrogen Life Technology, Carlsbad, CA, USA). Total RNA was reverse-transcribed to cDNA using ReverTraAce (TOYOBO, Tokyo, Japan) as instructed. Quantitative real-time PCR were performed by standard methods using species-specific primers (Table 1). Species-specific primers were designed according to the sequences of human (Nqo1, SOD2 and Gpx1) or mouse (mNqo1, mG6pdx, mSOD2, Gpx1 and mCat) origin. 18S rRNA expression levels in human and mouse cells were amplified by universal primers and used for the calibration of real-time RT-PCR.

**Table 1:** Primers used for quantitative Real-time PCR.

Gene	Primer direction	Sequences
Nqo1 (NM_000903.2)	Forward	5’-CCTTCCGGAGTAAGAAGGCA-3’
	Reverse	5’-CTGGAGTGTGCCCAATGCTAT-3’
SOD2 (NM_000636.3)	Forward	5’-GTTGGGGTTGGCTTGGTTT-3’
	Reverse	5’-GCGTGCTCCCACACATCA-3’
Gpx1 (NM_000581.3)	Forward	5’-CGTCCCTCTGAGGCACCA-3’
	Reverse	5’-CCGGACGTACTTGAGGGAA-3’
mNqo1 (NM_008706.5)	Forward	5’-GGGACATGAACGTCATTCTCTG-3’
	Reverse	5’-GGTCTCCTCCCAGACGGTTT-3’
mG6pdx (NM_008062.2)	Forward	5’-GCTGCACAAGATTGATCGAGAA-3’
	Reverse	5’-GGTACCCTCGTACTGGAAGCC-3’
mSOD2 (NM_013671.3)	Forward	5’-GCAAGGTCGCTTACAGATTGC-3’
	Reverse	5’-GCTTTCAGATAGTCAGGTCTGACG-3’
mGpx1 (NM_008160.6)	Forward	5’-ATGAACGATCTGCAGAAGCGT-3’
	Reverse	5’-GTCGGACGTACTTGAGGGAATT-3’
mCat (NM_009804.2)	Forward	5’-AGAGGAAACGCCTGTGTGAGA-3’
	Reverse	5’-CTTCTCAGCGTTGTACTTGTCCA-3’
18S rRNA (NR_003278)	Forward	5’-GGTCATAAGCTTGCGTTGATTAAG-3’
	Reverse	5’-CTACGGAAACCTTGTTACGACTTT-3’

### Hematoxylin-Eosin (H&E) staining

Twenty-four hours after APAP administration, the mice were sacrificed and small pieces of liver were fixed in 4% paraformaldehyde overnight. Then, the tissues were dehydrated with a sequence of ethanol solutions, embedded in paraffin wax and sectioned at a thickness of 5 μm thickness. Finally, tissue section was stained with H&E. All histopathological changes were observed under light microscopy (Olympus, Japan).

### TUNEL staining

Hepatocytes were labeled *in situ* using a TUNEL apoptotic detection kit (Roche Diagnostics Gmbh, Mannheim, Germany) per the manufacturer’s instructions. The slides of paraffin-embedded liver slices were deparaffinized and rehydrated, and the slides were covered entirely with 3% H_2_O_2_ for 15 min at room temperature to inactivate the endogenous peroxidases. The slides were washed in PBS three times (10 min each), and covered with 10% Tirtox-100 for 8 min, followed by the incubation with TUNEL reaction mixture at 37°C in a humidified atmosphere in the dark for 1 h. 50 μl converter-POD was added onto the tissues for a reaction at 37°C for 30 min. Then the samples were spotted with DAB fluid and hematoxylin. Finally, a light microscope (Olympus, Japan) was used for the observation. A dark brown DAB signal indicates positive staining (apoptotic cells). At least 1000 cells (TUNEL-positive cells and TUNEL-negative cells) were counted in each of eight separate low-power fields for each sample, and the percentage of TUNEL-positive cells was calculated.

### Statistical analyses

All statistical analyses were performed with the GraphPad Prism software. Values are expressed as the mean ± SEM. Pair-wise comparisons were performed with Student’s t-test (two-tailed) and multiple-group comparisons were performed with one-way ANOVA with Bonferroni’s post hoc test. A *p*-value < 0.05 was considered to be significant.

## Results and discussion

### FMN greatly enhances cellular antioxidant defense

**Fig 1:**
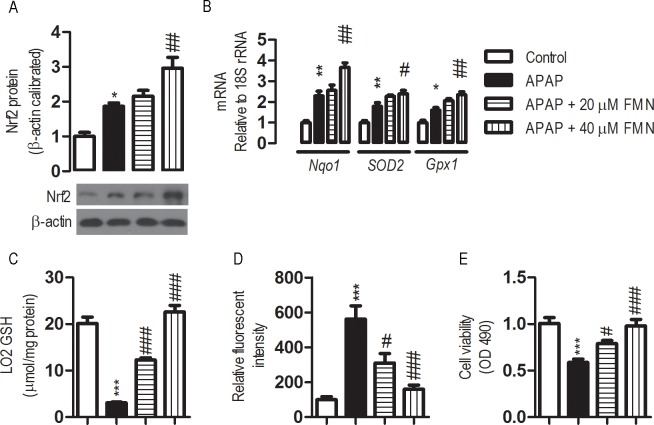
Effects of FMN on antioxidant defense in LO2 cells. Human non-tumor hepatic LO2 cells were maintained in DMEM medium supplemented with 10% FBS and incubated in a humidified incubator at 37°C in 5% CO2. The cell was initiated by replacing old media with a serum-free medium supplemented with or without FMN (20, 40 μM) for 6 h. Then, the cells were further incubated with APAP 10 mM for another 24 h. Cells collected were washed twice with PBS and used for GSH or cell viability analyses. (*) the APAP exposure *vs* control; (#) APAP+ 20 μM or APAP+ 40 μM *vs* the APAP exposure. Values represent the means ± SE; *n* = 4. #, *p* < 0.05; *** or ###, *p* < 0.001. (A) Effects of FMN on Nrf2 expression in LO2 cells. (B) Effects of FMN on mRNA expression of antioxidant enzymes in LO2 cells, as determined by quantitative real-time RT-PCR. (C) Effect of FMN on GSH levels in LO2 with 10 mM APAP exposure. (D) Effect of FMN on the content of ROS in LO2 with 10 mM APAP exposure. (E) Effect of FMN on cell viability in LO2 with 10 mM APAP exposure.

Oxidative stress is crucial to APAP-induced hepatocellular toxicity. Hepatic LO2 cells were treated with FMN to explore the effects of FMN on hepatocellular antioxidant defense. The expression of Nrf2 and several important antioxidant defensive enzymes were analyzed. It has been established that APAP stimulates Nrf2 and this is verified in our experiment (Fig 1A). Consistent with the up-regulation of Nrf2 protein by APAP exposure, the level of Nrf2 protein in FMN (40 μM) pre-treated cells exposed to APAP was ~160% higher than that of the cells exposed to APAP. This suggested that there is an APAP-independent stimulation on Nrf2 expression by FMN. Real-time RT-PCR analyses revealed that FMN profoundly affected the expression of antioxidant enzymes after APAP treatment. 10 mM of APAP exposure led to various degrees of induction in the mRNA expression for NAD(P)H quinone dehydrogenase 1 (*Nqo1*), Mn-superoxide dismutase (*SOD2*) and glutathione peroxidase 1 (*Gpx1*). In the presence of APAP, levels of expression for the tested enzymes in the FMN (40 μM) pre-treated cells were significantly higher than those of the control cells (APAP exposure only), suggesting a significant stimulation of antioxidant defense as a consequence of FMN treatment (Fig 1B).

Paralleling the up-regulation of the antioxidant properties, APAP-induced depletion of GSH and APAP-induced increase in ROS were significantly inhibited with pre-treatment of FMN (Fig 1C and 1D). Furthermore, cell viability was greatly increased in FMN pre-treated cells after APAP exposure (Fig 1E). FMN pre-treatment, therefore, might protect hepatic cells from APAP-induced toxicity, in part through enhancing hepatocellular antioxidant defense.

### FMN opposes APAP-induced hepatotoxicity in mice

**Fig 2:**
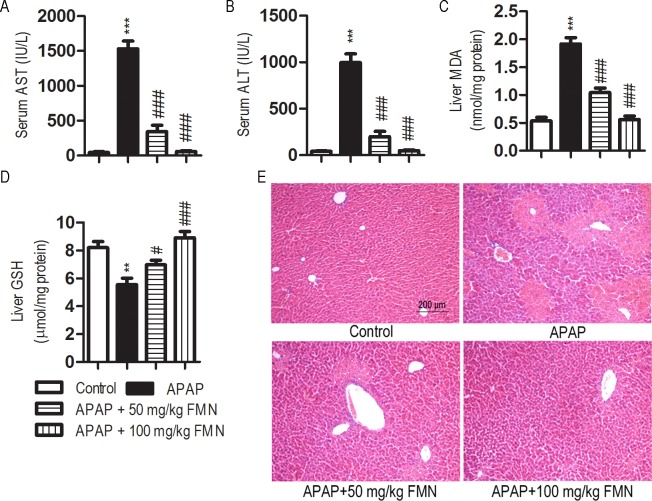
FMN protects against liver injury in APAP-overdose mice. (A, B) Serum AST and ALT levels in control group, APAP group, APAP + 50 mg/kg FMN group and APAP + 100 mg/kg FMN group. (C, D) Hepatic MDA and GSH contents in control group, APAP group, APAP + 50 mg/kg FMN group and APAP + 100 mg/kg FMN group. (E) Representative photomicrograph of H&E stained liver tissues from control group, APAP group, APAP + 50 mg/kg FMN group and APAP + 100 mg/kg FMN group. Values represent the means ± SE; *n* = 4–6. (*) the APAP group *vs* control; (#) APAP+ 50 mg/kg or APAP+ 100 mg/kg *vs* the APAP group. #, *p* < 0.05; ** or ##, *p* < 0.05; *** or ###, *p* < 0.001.

To further investigate the role of FMN in APAP-induced hepatotoxicity, we tested whether FMN can protect mice from APAP-induced hepatic injury *in vivo*. Serum levels of AST and ALT were detected as measures of liver function. Serum AST increased significantly in APAP group when compared with the control group (1533.8 ± 244.8 *vs* 44.9 ± 22.6 IU/L, *p* < 0.001). Serum AST levels were significantly lower in the APAP + 50 mg/kg FMN and APAP + 100 mg/kg FMN group than that in the APAP group (344.3 ± 197.2 and 56.7 ± 28.8 IU/L, *p* < 0.001). Serum ALT significantly increased in APAP group when compared with the control group (955.8 ± 209.4 *vs* 40.5 ± 14.9 IU/L, *p* < 0.001). Serum ALT levels were significantly lower in the APAP + 50 mg/kg FMN and APAP + 100 mg/kg FMN groups than that in the APAP group (197.2 ± 130.1 and 46.9 ± 20.4 IU/L, *p* < 0.001) (Fig 2B). There was no statistically significant difference in serum AST and ALT between the APAP + 100 mg/kg FMN group and the control group (Fig 2A and 2B).

Hepatic MDA and GSH levels were also determined, as good indicators of APAP-induced tissue damage. Hepatic MDA significantly increased in the APAP group when compared with the control group (1.91 ± 0.27 *vs* 0.53 ± 0.13 nmol/mg protein, *p* < 0.001). They were significantly decreased in the APAP + 50 mg/kg FMN and APAP + 100 mg/kg FMN groups when compared with the APAP group (1.04 ± 0.16 and 0.55 ± 0.14 nmol/mg protein, *p* < 0.001) (Fig 2C). At the same time, hepatic GSH was significantly decreased in the APAP group when compared with the control group (5.54 ± 0.93 *vs* 8.19 ± 0.88 μmol/mg protein, *p* < 0.01). There were significantly restored in the APAP + 50 mg/kg FMN and APAP + 100 mg/kg FMN groups when compared with the APAP group (6.96 ± 0.72 and 8.91 ± 1.11 μmol/mg protein, *p* < 0.05) (Fig 2D). There was no statistically significant difference in hepatic MDA and GSH contents between the APAP + 100 mg/kg FMN group and the control group (Fig 2C and 2D).

Liver sections from the APAP group stained with H&E showed features typical of inflammatory hepatic tissue, including centrilobular necrosis, sinusoidal congestion, lymphocytes infiltration and kupffer cells around the central vein, loss of cell boundary and ballooning degeneration, confirming the hepatic damage indicated by biochemical and enzymatic assays. However mice treated with FMN preserved normal hepatic architecture with minimal changes. Furthermore, liver section from the APAP + 100 mg/kg FMN group was similar to the appearance of liver tissue from the control group (Fig 2E).

### FMN prevents hepatocyte apoptosis

**Fig 3:**
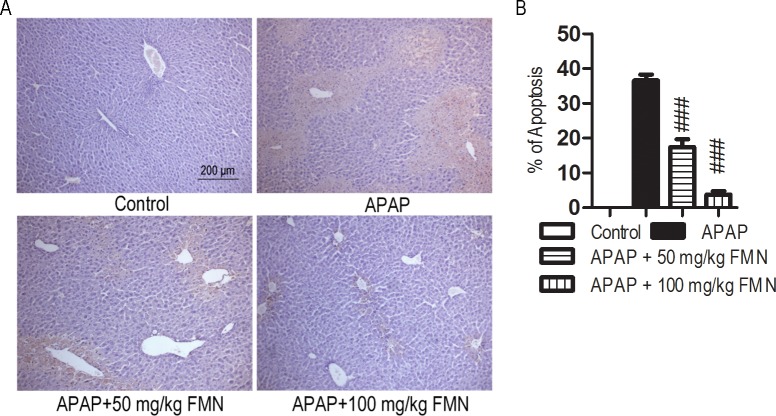
FMN prevents hepatocyte apoptosis induced by APAP. (A) Representative photomicrograph of TUNEL stained liver tissues from control group, APAP group, APAP + 50 mg/kg FMN group and APAP + 100 mg/kg FMN group. (B) TUNEL-positive cells were counted and the percentage of positive cells is illustrated. Values represent the means ± SE; *n* = 4–6. (#) APAP+ 50 mg/kg or APAP+ 100 mg/kg *vs* the APAP group. ###, *p* < 0.001.

Given the importance of apoptosis in APAP-induced liver injury, TUNEL assay was used to determine the extent of hepatocyte apoptosis. As shown in Fig 3A, there was massive hepatocyte apoptosis in the livers of mice treated with APAP. The apoptosis was significantly decreased in the APAP + 50 mg/kg FMN (15.92 ± 0.43%) and APAP + 100 mg/kg FMN (2.58 ± 1.8%) groups when compared with the APAP group (36.55 ± 3.82%, *p* < 0.001) (Fig 3A and 3B).

### FMN enhances Nrf2 and antioxidant gene expression

**Fig 4:**
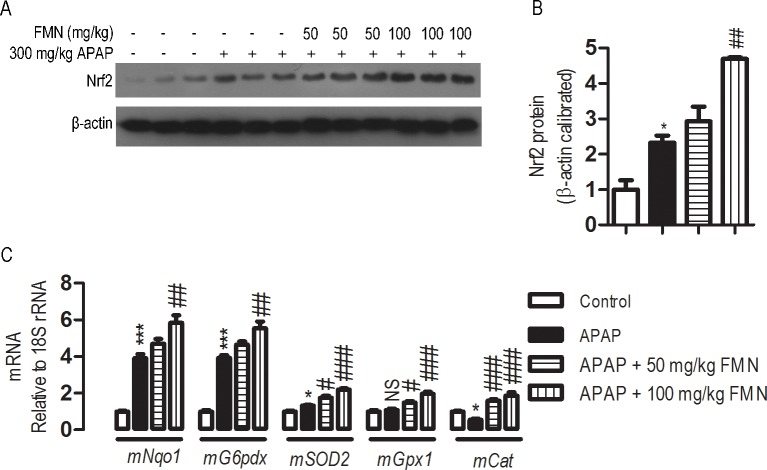
Effects of FMN on hepatic expression of Nrf2 and antioxidative genes. (A, B) The expression of Nrf2 protein in mice liver, as determined by western blot. β-actin was calibrated. (C) Nqo1, G6pdx, SOD2, Gpx1 and Cat mRNAs expression. mRNA expression was determined by real-time PCR. 18S rRNA was calibrated. Values represent the means ± SE; *n* = 4. (*) the APAP group *vs* control; (#) APAP+ 50 mg/kg or APAP+ 100 mg/kg *vs* the APAP group. NS, not significantly; #, < 0.05; ** or ##, *p* < 0.05; *** or ###, *p* < 0.001.

Based on the above observations, we further explore the possible mechanism by which FMN protects liver injury induced by APAP. Oxidative stress is central to APAP-induced hepatotoxicity. Nrf2 plays an important role in the activation of the antioxidant defense of APAP-induced liver injury. To evaluate whether Nrf2 confers protective effects of FMN against APAP-induced hepatotoxicity, Nrf2 protein expression was detected in mice liver. It is known that Nrf2 protein expression is up-regulated by APAP treatment in liver tissue, and this was verified in Fig 4A. It seemed to slightly increase in Nrf2 protein expression in the APAP + 50 mg/kg FMN group when compared with the APAP group, even though there was no statistical difference. However, the Nrf2 protein expression was significantly increased by 2.01-fold in the APAP + 100 mg/kg FMN group when compared with the APAP group (*p* < 0.01) (Fig 4A and 4B). Furthermore, a number of important antioxidant gene expressions were analyzed by real-time PCR. In mice exposed to APAP, significant elevations in mRNA expression were observed for *mNqo1*, Glucose-6-phosphate dehydrogenase X-linked (*mG6pdx*), *mSOD2*, whereas *mGpx1* remained unchanged, and Catalase (*mCat*) was down-regulated, in comparison with the control group. Levels of expression for *mNqo1*, *mG6pdx*, *mSOD2*, *mGpx1* and *mCat* in the APAP + 50 mg/kg FMN and APAP + 100 mg/kg FMN groups were significantly higher than that in the APAP group, except that for *mNqo1* and *mG6pdx* in the APAP + 50 mg/kg FMN group (Fig 4C).

FMN is one of the major isoflavonoid constituents from red clover, which possesses anti-inflammatory, antioxidant and anti-apoptosis activity. However, potential hepatoprotective effects and the possible involvement of Nrf2 and antioxidant properties as the underlying mechanism have not been investigated. To our knowledge, the present study for the first time showed that pretreatment of FMN has potential hepatoprotective effects, as evidenced by the significant inhibition of APAP-induced hepatotoxicity *in vitro* and *in vivo*. In human non-tumor hepatic cells LO2, the pre-treatment of FMN significantly enhanced Nrf2 protein expression, stimulated mRNA expression of antioxidant enzymes, and restored GSH level and cell viability upon APAP exposure. In mice, FMN significantly inhibited APAP-induced changes in liver biochemical parameters, lipid peroxidation products, GSH levels, histopathology and apoptosis. We further showed that Nrf2 and antioxidant properties may, at least in part, elucidate the underlying mechanisms.

Hepatocellular injury caused by a high dose of APAP results in the release of ALT and AST into the extracellular space, which are present in hepatocyte cytoplasm under normal condition[20]. The estimation of ALT and AST is a useful quantitative marker of the extent of hepatocellular damage[20]. ALT and AST were significantly elevated in the APAP group in comparison with the control group. Treatment with FMN protected the liver against APAP-induce hepatocellular injury, as evidenced by the decrease in serum ALT and AST activity. This observed protective effect might be a consequence of the improvement of the potential mechanisms by which APAP cause hepatocellular damage, with subsequent inhibition of the leakage of ALT and AST into the serum.

The protective effect of FMN against APAP-induced liver injury was confirmed by histopathology observation. The histopathological analysis of liver section showed normal configuration with distinct hepatic cell, sinusoidal spaces, and central veins in control group. APAP challenge provoked apparent histopathological changes, such as centrilobular hepatic necrosis and the presence of infiltrating lymphocytes. It has been observed previously that APAP overdose causes ultrastructural change in liver tissue. Following FMN administration, as shown in Fig 2E, the sections of liver tissues preserved their normal architecture, especially in the APAP + 100 mg/kg FMN group. Consistent with serum ALT and AST analysis, FMN can prevent APAP-induced hepatocellular damage, thus preserving the liver morphology.

Oxidative stress has been suggested to play a critical role in APAP-induced hepatocellular toxicity, and compounds with protective effect against APAP-induced hepatotoxicity have been described with antioxidant activity[21, 22]. The toxicity is mainly mediated by the covalent binding of NAPQI, the reactive metabolite of APAP, to sulfhydryl groups of GSH, other cellular protein (such as mitochondrial protein) and their subsequent oxidation. Overproduction of free radicals in hepatic cells may trigger lipid peroxidation, impair mitochondrial respiration, and interferes with calcium homeostasis, thereby inducing hepatic cell death and liver failure[22]. A rapid depletion of GSH and lipid peroxidation has been reported in liver of animals treated with high dose of APAP. This may explain the decreased GSH contents in LO2 and in experimental mice liver which combat the increased formation of free radicals. Moreover, we demonstrated that APAP exposure significantly increased MDA levels. The MDA is a good biomarker of the extent of lipid peroxidation, which is a well-established mechanism of cellular injury[23]. Administration of FMN significantly ameliorated the APAP-induced increase in MDA levels and decreased the GSH contents toward normal values. APAP-induced apoptosis was observed not only in cultured hepatic cells, but also in livers of mice treated with overdose of APAP. Accumulating evidence implicates that hepatocyte apoptosis plays an important role in APAP-induced hepatic damage, although the actual event resulting in cell death is still controversial. Extensive hepatocyte apoptosis was observed in the liver of the APAP group, and APAP exposure decreased cell viability in LO2. This result suggested that APAP-induced apoptosis contributes to the repression of cell viability in LO2. FMN treatment significantly prevented the apoptosis induced by APAP in experimental mice and restored the cell viability in LO2.

The antioxidant defense system is often activated as a compensatory response to provide the protection for cell damage upon oxidative challenge. Our present study showed that overdose of APAP for 24 h induces Nrf2 protein and some antioxidant enzymes mRNA expression in experimental mice. The nuclear factor Nrf2 is a transcription factor essential for activation of the antioxidant defense system, which has been shown to be actively involved in protecting hepatic cells from APAP overdose-induced oxidative in mice. Upon activation by oxidants, Nrf2 translocates from cytosol to nuclei, where it is recruited to the antioxidant response element (ARE) in the regulatory region of its target genes as a heterodimer with small Maf proteins. Nrf2 knockout mice are more sensitive to APAP-induced hepatotoxicity compared to WT littermates. In contrast, activation of hepatic Nrf2 by a genetic method or by chemical reagents confers mice with significant resistance to APAP-induced liver damage by inducing the expression of antioxidant genes. Surprisingly, Nrf2 protein expression was significantly up-regulated in the liver in the APAP + 100 mg/kg FMN group. Antioxidant gene mRNA expression, *mNqo1*, *mG6pdx*, *mSOD2*, *mGpx1 and mCat*, was markedly increased in the APAP + 100 mg/kg FMN group in comparison with the APAP group. m*Nqo1*, *mG6pdx*, *mSOD2* are reported as the direct transcriptional targets of Nrf2. These results provide strong evidence supporting the hypothesis that FMN-mediated up-regulation of antioxidant genes is attributable, in part to enhanced Nrf2 expression. It is not clear whether m*Gpx1* and m*Cat* are regulated by Nrf2. However, *mCat* mRNA expression markedly decreased and there was no statistically change in *mGpx1* mRNA expression after administration of overdose of APAP for 24 h in experimental mice. *mGpx1* and *mCat*, were markedly increased in the APAP + 50 mg/kg FMN and APAP + 100 mg/kg FMN group when compared with the APAP group. Previous studies have provided evidence that FMN exhibits concentration-dependent inhibition of CYP1A2 which is a member of CYP450 in human and mice liver microsomes[24]. As APAP is metabolized by CYP450 enzymes in microsomes, it was expected that CYP450-mediated metabolism of APAP generated ROS in APAP-induced hepatotoxicity, leading to subsequent lipid peroxidation and liver injury. It could therefore be postulated that inhibition of CYP450 activity by FMN ameliorates the hepatic oxidative stress and NAPQI production and leads to improved liver damage after APAP overdose. The results implicated that, in addition to up-regulation of Nrf2, there are other mechanisms contributing to hepatoprotection by FMN in APAP-induced liver injury.

## Conclusions

In summary, our present study revealed that FMN has a protective effect on APAP-induced hepatocellular toxicity in LO2 and in experimental mice. The hepatic cell protection effect by a decrease of lipid peroxidation, reduced liver injury, and restore hepatocellular GSH, at least in part through enhancing Nrf2 and antioxidant genes expression.
